# Krebs von den lungen-6 as a clinical marker for hypersensitivity pneumonitis: A meta-analysis and bioinformatics analysis

**DOI:** 10.3389/fimmu.2022.1041098

**Published:** 2022-11-30

**Authors:** Jie He, Jiangliu Zhang, Xinyi Ren

**Affiliations:** ^1^ Clinical Medical College of Chengdu Medical College. Chengdu, Sichuan, China; ^2^ Department of Pulmonary and Critical Care Medicine, The First Affiliated Hospital of Chengdu Medical College, Chengdu, Sichuan, China

**Keywords:** Krebs von den Lungen-6, hypersensitivity pneumonitis, systematic review, meta-analysis, bioinformatics analysis

## Abstract

**Aim:**

Hypersensitivity pneumonitis (HP), also referred to as exogenous allergic alveolitis, is one of the most common interstitial lung diseases (ILDs). A potential immune biomarker, Krebs von den lgen-6 (KL-6) characterizes the progression and severity of HP. The meta-analysis in this study was conducted to elucidate the variations in the concentrations of KL-6 in different types of HP.

**Methods:**

A systematic search of various databases such as EMBASE, Pubmed, CNKI, VIP, Web of Science, and WanFang was carried out to find relevant published articles between January 1980 and August 2022 that explored the relationship between KL-6 and allergic pneumonia. Standardized mean difference (SMD) and 95% confidence interval (CI) were used as effect sizes for comparison among different groups. The GSE47460 and GSE150910 datasets were downloaded to extract and validate the differences in KL-6 mRNA expression between HP lung tissue and healthy controls. Furthermore, the single-cell sequencing dataset GSE135893 was downloaded to extract KL-6 mRNA expression in type II alveolar epithelial cells to validate the differences between HP and healthy controls. Two researchers evaluated the quality of the included studies by employing Newcastle-Ottawa Scale. All the qualified studies were subjected to statistical analyses carried out utilizing RevMan 5.2, Stata 11.0, and R software 4.1.3.

**Results:**

Twenty studies aligned perfectly with the inclusion criteria of the meta. The concentrations of KL-6 were substantially higher in the blood of HP patients as compared to the control group. Subgroup analyses were carried out in accordance with the allergen source and the results revealed that patients with different allergens had higher blood KL-6 concentrations than healthy controls. Additionally, different subgroups of subjects were created for meta-analysis as per the fibrosis status, race, measurement method, and sample type. The concentration of KL-6 in blood was much higher in all HP subgroups than in healthy control groups. Moreover, the bioinformatics analysis revealed that KL-6 mRNA expression was higher in HP lung tissue and type II alveolar epithelial cells as compared to healthy controls.

**Conclusion:**

The present meta-analysis and bioinformatics analysis suggested that the concentration levels of KL-6 varied between HP patients and healthy individuals, and the KL-6 concentrations may be higher in the blood samples of HP patients.

**Systematic review registration:**

https://www.crd.york.ac.uk/prospero/, CRD42022355334.

## 1 Introduction

Hypersensitivity pneumonitis (HP) is an interstitial lung disease (ILD) caused by immune-mediated exposure of individuals that are susceptible to allergens in the environment. Common allergens include microorganisms (bacteria, fungal spores), animal proteins (pigeon feathers, feces), chemical sensitizers (isocyanates), and organic dust particles (1). The incidence of HP varies according to climate, occupation, and environment. An epidemiological survey conducted in Denmark indicated that the annual incidence of HP from 1998-2010 was about 1.16/100,000 (2), and a prevalence survey conducted in the USA found that the frequency of HP was about 1.67-2.71/100,000, and a cumulative annual incidence of 1.28-1.94/100,000 (3) from 2004-2013. Similarly, in the United Kingdom, an epidemiological survey conducted on the incidence of occupational hypersensitivity pneumonitis (OHP) from 1996-2015 showed an annual incidence rate of approximately 1.4 per 1 million (4). Barnes et al. (5) summarized in 2022 that the incidence of HP in high-risk populations was estimated to be higher, ranging from 1.3-12.9% in farmers, 3.7%-10.4% in bird breeders, and 3.5%-29% in mushroom workers. The clinical manifestations of HP are diverse, and the duration of the disease varies. The actual incidence of the disease might be much higher than that reported in the literature. Previously, HP was classified as acute, subacute, or chronic according to the duration of the disease, but the exact time of definition has not been standardized (6). An update of the diagnostic guidelines for adult allergic pneumonia was developed and published jointly by the American Thoracic Society, the Japanese Society of Respiratory Diseases, and the Latin American Thoracic Society on August 1, 2020, which classifies HP into non-fibrotic and fibrotic types depending on the presence or absence of fibrotic features on imaging or histopathology. The pathogenesis of HP may be related to type III and type IV hypersensitivity reactions (7), but the exact pathogenesis is not known. Therefore, there is a lack of efficient and simple serologic correlates to aid in diagnosis and disease assessment.

Krebs von den Lungen-6 (KL-6) is a glycoprotein localized on the membrane of type II alveolar epithelial cells, mainly released by propagating or damaged type II alveolar epithelial cells, and is specific for determining damage to these cells (8). High expression of KL-6 concentrations may be associated with allergic pneumonia. Silvia Sánchez-Díez et al. (9) studied the expression concentrations of KL-6 in 34 individuals with fibrotic HP (fibrotic hypersensitivity pneumonitis, fHP) in comparison to 15 patients with non-fibrotic HP (nfHP). Elevated expression concentrations of serum KL-6 were detected in fHP patients as compared to nfHP patients. Moreover, the concentrations of serum KL-6 displayed a negative association with TLC (total lung capacity) and diffusing capacity of the lungs for carbon monoxide. However, additional verification of the results is required because of the small sample size of the individual studies.

Therefore, a meta-analysis was essential for the quantitative evaluation of the correlation between KL-6 concentration and the occurrence of HP by utilizing all available relevant studies. The microarray data and single-cell sequencing results were included in this study by means of biological analysis to further validate the reliability of the meta-analysis results.

## 2 Material and methods

### 2.1 Search strategy and literature selection

The meta-analysis conducted in this study was registered in the International Prospective Register for Systematic Evaluation (PROSPERO, https://www.crd.york.ac.uk/PROSPERO/, ID isCRD42022355334). One investigator comprehensively searched published research articles from January 1980 to August 2022 in Pubmed, EMBASE, CNKI, VIP, Web of Science, and WanFang databases for the search terms “allergic pneumonia” or “exogenous allergic alveolitis” or “hypersensitivity pneumonitis” or “Exogenous allergic alveolitis “ or “HP” or “EAA” and “salivary liquefied glycoconjugate antigen 6” or “Krebs von den Lungen-6” or “KL-6”.

### 2.2 Study selection and exclusion criteria

Inclusion criteria (1): case-control studies, cross-sectional studies, or cohort studies (2); literature studies involving KL-6 level in blood samples from patients with HP, including individuals with acute hypersensitivity pneumonitis (AHP), chronic allergic pneumonia, chronic hypersensitivity pneumonitis (CHP), fibrotic hypersensitivity pneumonitis (fHP) and non-fibrotic hypersensitivity pneumonitis (nfHP); the content of the literature was not limited by age, sex, nationality or race (3); HP diagnostic criteria according to the guidelines established by the American Thoracic Society (ATS)/Japanese Respiratory Society (JRS)/Latin American Thoracic Society (ALAT) in 2020 (10), i.e., a clear history of environmental antigen exposure; the corresponding clinical signs of HP (cough, dyspnea, fever, etc.) or improvement after removal from environmental antigen exposure; chest imaging suggestive of interstitial lung disease (diffuse ground glass shadow, patchy shadow, central lobar nodular shadow, mosaic sign, grid shadow or with cellular lung in both lungs); pulmonary function tests suggestive of restrictive pulmonary ventilation dysfunction and diffusion dysfunction; alveolar lavage fluid examination suggestive of increased lymphocytes and/or histopathological examination suggesting non-necrotizing granuloma, lymphofollicular fine bronchitis, and interstitial pneumonia, with the exception of diseases such as nodular disease, interstitial lung changes secondary to connective tissue disease, and idiopathic pulmonary fibrosis (4); complete data in the literature or the data required could be extrapolated from known results (5); full-text articles in English and Chinese (7); for repeatedly published studies, reports with larger sample size or more detailed information were selected.

Exclusion criteria (1): inability to obtain sufficient data from the original research article and failure to communicate with the authors to obtain detailed data (2); case reports, review articles, meta-analyses, conference papers, and animal studies; and (3) articles with overlapping data already included in this study.

### 2.3 Literature screening, data extraction and quality evaluation

Based on the above search method, an initial screening of eligible articles based on article titles and abstracts was performed by two researchers. Subsequently, the re-evaluation of the selected articles was carried out by reading and reviewing the full text carefully. If there was disagreement between the two researchers, a third investigator was contacted and a final unanimous decision was made after consultation.

### 2.4 Data extraction and management

Tables were created to retrieve the following information from articles eligible for inclusion (1): basic information: first author, publication date, etc. (2); baseline characteristics of study participants: e.g., country, race, age, sample size, study design, measurement method, measurement sample type, allergens causing HP, and KL-6 concentration of the subject.

### 2.5 Quality assessment

The literature’s quality was evaluated by two researchers following the Newcastle-Ottawa Scale (NOS) (11). In case of disagreement during the quality assessment process, two reviewers were consulted, or an expert in the field arbitrated. Each literature was given a maximum score of 9, including study population selection (4 out of 4 items), comparability (1 out of 2 items), and exposure or outcome (3 out of 3 items), with 0 to 3, 4 to 6, and 7 to 9 indicating low, moderate and high quality, respectively.

### 2.6 Bioinformatics analysis

Data sets (GSE47460, GSE150910, and GSE135893) needed for bioinformatics analysis were obtained from the Gene Expression Omnibus database (GEO) (https://www.ncbi.nlm.nih.gov/geo/). GSE47460 (12) microarray data containing 30 HP and 108 normal lung tissue cases, is based on GPL6480 and GPL14550 platform (Agilent-014850 Whole Human Genome Microarray 4x44K G4112F; Agilent-028004 SurePrint G3 Human GE 8x60K Microarray). GSE47460 also contains DLCO% predicted values (diffusing capacity for carbon monoxide) and FEV1% predicted values for 30 HP patients. The GSE150910 data (13) encompassing 82 HP and 103 normal lung tissue samples, is based on the GPL24676 platform (Illumina Nova Seq 6000, *Homo sapiens*). Dataset GSE135893 (14), a single-cell sequencing dataset, contains sequencing data from 3 HP lung tissues and 10 normal lung tissues (Illumina NextSeq 500, Illumina HiSeq 4000, Illumina NovaSeq 6000, *Homo sapiens*).

The “GEOquery” software was utilized to obtain the original data that was evaluated by means of the oligo software package in R software (version 4.1.3). Subsequently, the manufacturer-provided annotation file was followed to change the name of the probe to a gene name, and the probes lacking gene names were removed. The “affy” software package of the R software was employed for the chip dataset’s preprocessing and normalization. The gene expression quantifications of GSE47460 were corrected for batches using ComBat from the “sva” R package. The R package “Biomart” was utilized for the filtration and validation of the GSE150910 data set by removing duplicated and missing data and transforming it by employing the statistical procedure log2 (TPM).

The GSE135893 dataset’s (lung) computational analysis was carried out by employing the R package “Seurat” (4.0.3). Quality control analyses (200 < number of feature RNA < 5,000, percentage of mitochondrial genes < 20%, percentage of ribosomal genes > 3, and percentage of erythrocyte gene < 0.1) were carried out in both datasets as per the R package “Seurat”, respectively. The Seurat RunPCA function was utilized for the calculation of the principal component analysis (PCA). The scRNA-seq data was normalized by employing Seurat NormalizeData functions, while Seurat IntegrateData and FindIntegrationAnchors functions, established on robust principal component analysis (RPCA), were utilized for the integration of multiple samples. UMAP (uniform manifold approximation and projection) was employed for dimension reduction; and Louvain clusters were analyzed utilizing the 30 primary principal components with the Seurat FindClusters function and RunUMAP function, respectively, with the resolution adjusted at 0.8. Seurat’s FindAllMarkers function was employed to locate markers of clusters, and identification of cell types was conducted based on markers of each cluster in accordance with CellMarker (15), PanglaoDB databases (16), and the original research articles of both datasets. Visualization of the expression of genes and their distribution was carried out in accordance with Seurat’s VlnPlot, DotPlot, and FeaturePlot functions. The R software’s “select” function was employed to evaluate the KL-6 mRNA expression in the respective samples of the GSE47460, GSE150910, and GSE135893.

### 2.7 Statistical analysis

The subsequent data analyses were accomplished by employing Stata 11.0 (Stata Corporation, College Station, TX, USA) and the RevMan 5.2 software program (https://tech.cochrane.org/revman) to determine SMD and 95% CI, and the Z-test was employed to assess the impact of the combined SMD. Cochran Q and I^2^ indicators were employed to assess the heterogeneity in each study, with scores ranging from 0% to 100%. Additionally, substantial heterogeneity was witnessed in the illustration of P heterogeneity with P_heterogeneity_ < 0.1 and I^2^ > 50%. The combined SMD and 95% CI values were calculated by employing the fixed-effect and random-effect models. The sensitivity and meta-regression analyses were employed to calculate the heterogeneity sources, and subgroup analysis was carried out as per the type of allergen, fibrosis, race, assay approach, and sample types. The funnel diagram asymmetry was utilized to assess publication bias, and Egger’s and Begg’s test results were calculated by means of Stata 11.0 software. The values reported in terms of median and quartile in several articles were altered to mean and standard deviation (17, 18). The bioinformatics analysis was carried out to compare the KL-6 mRNA expression level between HP and control groups by using the Wilcoxon rank sum test (non-parametric). Pearson’s correlation analysis was utilized to calculate the link between mRNA expression levels of KL-6, DLCO% predicted values and FEV1% predicted values, where p<0.05 was considered a substantial difference. GraphPad Prism 8 (https://www.graphpad.com/scientific-software/prism/) and R software 4.1.3 were employed to carry out the visualization and various statistical analyses.

## 3 Results

### 3.1 Literature search and inclusion

**Figure 1:**
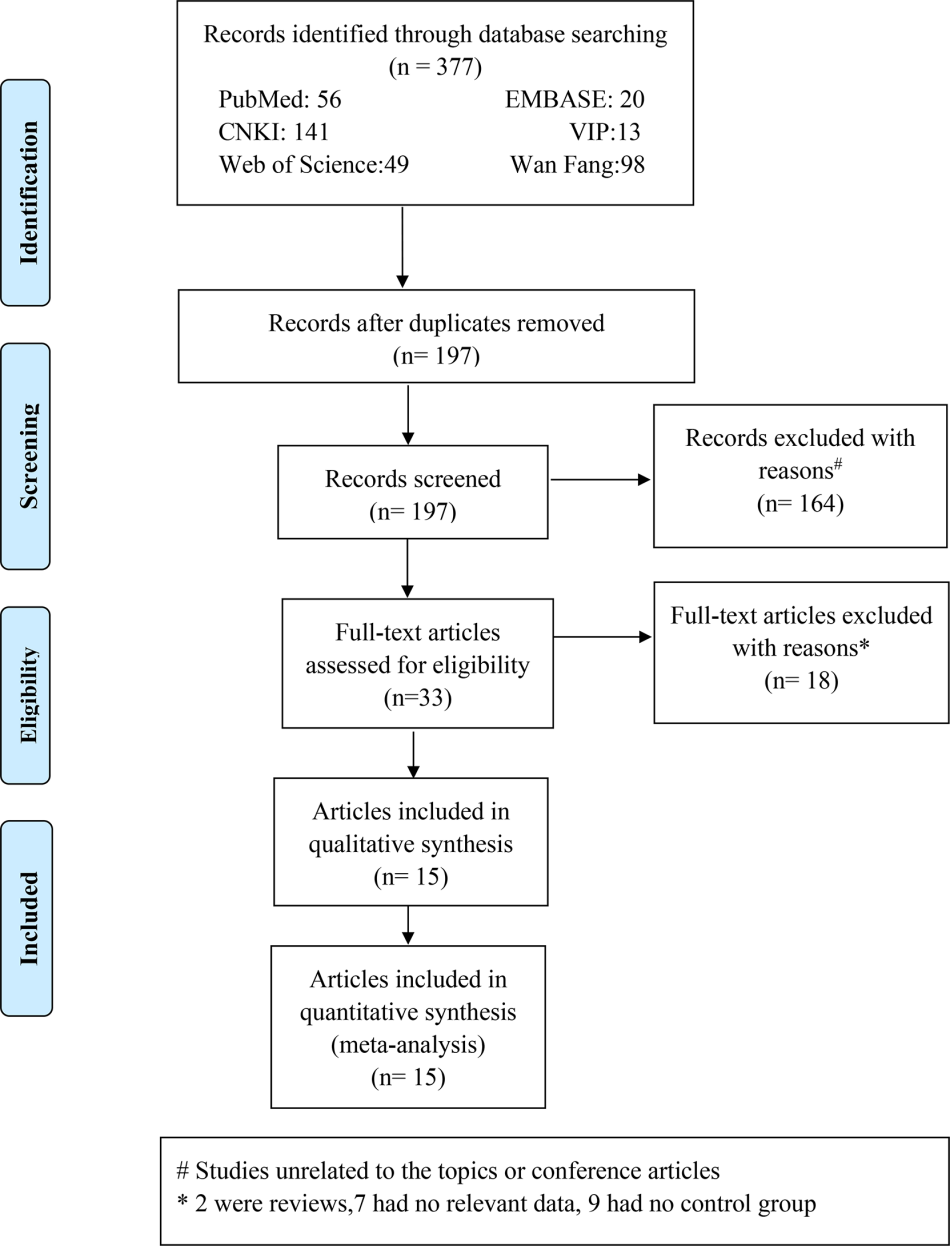
This flow chart illustrates the process of searching for literature and selecting studies.

A total of 377 Chinese and English research papers were retrieved for this study. The initial reading excluded duplicate studies leaving 197 articles. Further reading and review of titles and abstracts excluded 164 articles, which were unrelated to topics or conference articles. Yielding 33 articles were downloaded and reviewed thoroughly. A total of 18 research papers were eliminated for the reasons indicated in **Figure 1**. We retrieved the full texts of the articles and excluded several full texts for following the reasons: two were reviews; seven had no relevant data; nine had no control or control group. Finally, 15 articles were included, with 3 articles in Chinese (19–21) and 12 articles in English (9, 22–32). The fifteen articles involved twenty studies. The literature search process is detailed in **Figure 1**. Of these 20 studies, one study (32) mentioned that the case group included acute HP patients and the control group included chronic HP patients, and nineteen studies (9, 22–31) mentioned that the case group included HP patients and the control group included healthy participants.

### 3.2 Basic characteristics and quality of the included literature

The 15 included articles (9, 19–32) involved 641 patients with HP, 656 healthy individuals, 35 patients with acute allergic pneumonia, 57 chronic allergic pneumonia patients, 277 fibrosing allergic pneumonia patients, and 39 patients with non-fibrosing allergic pneumonia. The included literature’s basic information can be found in **Tables 1** and **2**.

**Table 1:** Characteristics of included studies.

Study	Years	Country	Ethnicity	Study design	Assay approach	Sample type	Allergen	NOS		Sample number(n) (Male/Female)	
										HP	Controls
d'Alessandro M	2020	Italy	Caucasian	CCS	ELISA	Serum	NA		7	34 (20/14)	22 (6/16)
d'Alessandro M	2021	Italy	Caucasian	CCS	ELISA	Serum	NA		7	14 (8/6)	12 (5/7)
Ding YJ	2018	China	Asian	CCS	CLEIA	Serum	NA		6	37 (21/16)	35 (20/15)
He XB	2005	China	Asian	CCS	NA	Serum	NA		6	16 (9/7)	20 (10/10)
Janssen R	2005	Netherlands	Caucasian	CCS	ELISA	Serum	Birds		7	49 (27/22)	38 (18/20)
Ji Y2020	2020	UK	Caucasian	CSS	ELISA	Plasma	Birds		7	20 (NA)	10 (NA)
McSharry CP	2006	UK	Caucasian	CSS	ELISA	Serum	Birds		6	55 (NA)	15 (NA)
Mostafa AI (fHP)	2021	Egypt	Caucasian	CCS	ELISA	Serum	NA		8	27 (NA)	20 (NA)
Mostafa AI (nfHP)	2021	Egypt	Caucasian	CCS	ELISA	Serum	NA		8	24 (NA)	20 (NA)
Mostafa AI (Bird -HP)	2021	Egypt	Caucasian	CCS	ELISA	Serum	Birds		8	35 (NA)	20 (NA)
Nukui Y	2019	Japan	Asian	CCS	ELISA	Serum	Birds		8	61 (33/28)	50 (30/20)
Okamoto T	2015	Japan	Asian	CCS	ELISA	Serum	NA		8	92 (44/48)	NA
Ren DH	2015	China	Asian	CCS	CLEIA	Serum	NA		6	149 (65/84)	64 (33/31)
Silvia Sánchez-Díez (fHP and Bird -HP)	2022	Spain	Caucasian	CSS	ELISA	Serum	Birds		8	24 (NA)	67 (30/37)
Silvia Sánchez-Díez (fHP and Fungi-HP)	2022	Spain	Caucasian	CSS	ELISA	Serum	Fungi		8	10 (NA)	67 (30/37)
Silvia Sánchez-Díez (nfHP and Bird-HP)	2022	Spain	Caucasian	CSS	ELISA	Serum	Birds		8	8 (NA)	67 (30/37)
Silvia Sánchez-Díez (nfHP and Fungi-HP)	2022	Spain	Caucasian	CSS	ELISA	Serum	Fungi		8	7 (NA)	67 (30/37)
Takahashi T	2000	Japan	Asian	CSS	ELISA	Serum	Thermophilic actinomycetes		7	5 (NA)	237 (NA)
Tsushima K	2005	Japan	Asian	CSS	NA	Serum	Mushroom		6	4 (NA)	22 (0/22)
Yoshikawa S	2007	Japan	Asian	CSS	CLEIA	Serum	Mushroom		7	5 (1/4)	44 (22/21)

CSS, cross sectional study; CCS, case–control study; NA, not applicable; HP, Hypersensitivity pneumonitis; fHP, fibrotic Hypersensitivity pneumonitis; nfHP, non fibrotic Hypersensitivity pneumonitis; AHP, Acute Hypersensitivity pneumonitis; CHP, Chronic hypersensitivity pneumonitis. ELISA, Enzyme linked-immuno-sorbent assay; CLEIA, chemiluminescence immunoassay assay; UK, The United Kingdom; NOS, Newcastle-Ottawa Scale.

**Table 2:** Participants’ characteristics of included studies.

Study	Years	Sample (n)	Smoking habits (n) (current/never/former)	Age (y)	KL-6
		HP	HP	HP	HP
		Controls	Controls	Controls	Controls
d'Alessandro M	2020	34	0/14/20	65 ± 9.5	1691 ± 1643 U/ml
		22	0/17/5	54 ± 14.9	268 ± 148 U/ml
d'Alessandro M	2021	14	0/6/8	65.2 ± 12.3	1047.7 ± 308.1 U/ml
		12	0/8/4	65.2 ± 12.3	189 ± 100.6 U/ml
Ding YJ	2018	37	NA	60.9 ± 6.4	1801.26 ± 185.36 U/ml
		35	NA	60.8 ± 6.4	201.17 ± 20.37 U/ml
He XB	2005	16	NA	62.3 ± 11.7	1218 ± 647 U/ml
		20	NA	60.5 ± 10.3	247 ± 120 U/ml
Janssen R	2005	49	2/21/26	56 ± 12	1396.6 ± 894.2 U/ml
		38	0/27/11	46 ± 14	207 ± 66 U/ml
Ji Y2020	2020	20	NA	49.8 ± 15.1	818.4 ± 674.9 U/ml
		10	NA	NA	266.6 ± 194.3 U/ml
McSharry CP	2006	55	0/55/0	53.5 ± 7.9	511.7 ± 261.8μg/mL
		15	0/15/0	49.3 ± 4.4	296.9 ± 230.6μg/mL
Mostafa AI(fHP)	2021	27	NA	NA	1,165.00 ± 283.0 IU/ml
		20	NA	NA	138.2 ± 69.5 IU/ml
Mostafa AI(nfHP)	2021	24	NA	NA	2020.83 ± 870.77 IU/ml
		20	NA	NA	138.2 ± 69.5 IU/ml
Mostafa AI(Bird-HP)	2021	35	NA	NA	1644.43 ± 835.71 IU/ml
		20	NA	NA	138.2 ± 69.5 IU/ml
Nukui Y	2019	61	NA	63.8 ± 11	1236 ± 1072.8 U/ml
		50	NA	47.3 ± 9.9	191 ± 70.2 U/ml
Okamoto T *	2015	35	19/13/3	58.6 ±15.4	3350 ± 3246 U/ml
		57	26/20/11	61.3 ± 9.9	1639.9 ± 1250.3 U/ml
Ren DH	2015	149	NA	58.8 ± 11.7	1801.86 ± 2831.36 U/ml
		64	NA	39 ± 13.9	201.28 ± 81.18 U/ml
Silvia Sánchez-Díez (Bird -HP)	2022	24	NA	NA	2805.6 ± 5346.9 U/ml
(fHP and Bird -HP)		67	0/0/67	NA	283.7 ± 282.6 U/ml
Silvia Sánchez-Díez (Fungi-HP)	2022	10	NA	NA	1590.3 ± 2604.1 U/ml
(fHP and Fungi-HP)		67	0/0/67	NA	283.7 ± 282.6 U/ml
Silvia Sánchez-Díez (fHP)	2022	8	NA	NA	1298.7 ± 2164.1 U/ml
(nfHP and Bird-HP)		67	0/0/67	NA	283.7 ± 282.6 U/ml
Silvia Sánchez-Díez (nfHP)	2022	7	NA	NA	1075.6 ± 1996.0 U/ml
(nfHP and Fungi-HP)		67	0/0/67	NA	283.7 ± 282.6 U/ml
Takahashi T	2000	5	NA	NA	1263 ± 644 U/ml
		237	NA	NA	207 ± 92.37 U/ml
Tsushima K	2005	4	NA	NA	981 ± 331 U/ml
		22	0/22/0	49.2 ± 7	251 ± 68 U/ml
Yoshikawa S	2007	5	0/4/1	54.5 ± 12	2790.4 ± 2607.6 U/ml
		44	NA	NA	265.8 ± 136.2 U/ml

(*: In this study, the case group was AHP and the control group was CHP.)

NA, not applicable; KL-6, Krebs von den Lungen 6; HP, Hypersensitivity pneumonitis; fHP, fibrotic Hypersensitivity pneumonitis; nfHP, non fibrotic Hypersensitivity pneumonitis; AHP, Acute Hypersensitivity pneumonitis; CHP, Chronic hypersensitivity pneumonitis.

### 3.3 Literature quality evaluation

The literature’s quality was evaluated by employing the Newcastle-Ottawa Scale scores (11). Every article in this study scored 6 or more, with four articles scoring 8, six articles scoring 7, and five articles scoring 6, indicating the relatively high quality of the included literature (**Table 1**).

### 3.4 Meta-analysis

#### 3.4.1 Variations in KL-6 concentration between HP patients and healthy individuals

**Figure 2:**
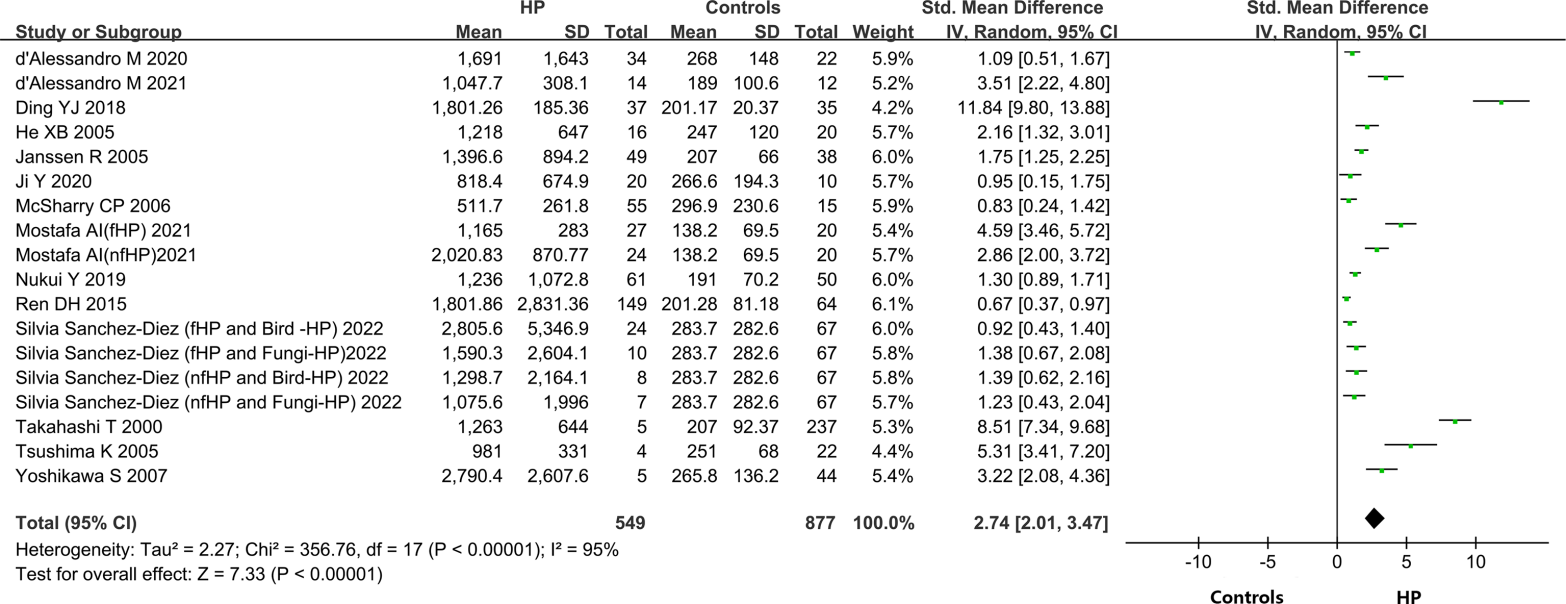
The forest plot pooled the SMD (95%) of KL-6 level between HP patients and controls.

A total of 14 out of the 15 included articles reported variations in KL-6 concentration between overall HP patients and healthy controls (9, 19–32). These 14 articles, involving 18 studies, contained 549 HP patients and 656 healthy individuals. Due to the heterogeneity between the results of the studies (I^2^ = 95%, P < 0.0001), a random-effects model was chosen for the combined analysis. Meta-analysis results indicated that overall HP patients had substantially higher expression of KL-6 than healthy controls (SMD = 2.74, 95% CI = 2.01-3.47, I^2^ = 95%, P < 0.0001) (**Figure 2**).

#### 3.4.2 Subgroup analysis

A subgroup analysis was carried out on the included literature because of the high heterogeneity of the meta-analysis of the overall population.

##### 3.4.2.1 Allergenic sources

**Figure 3:**
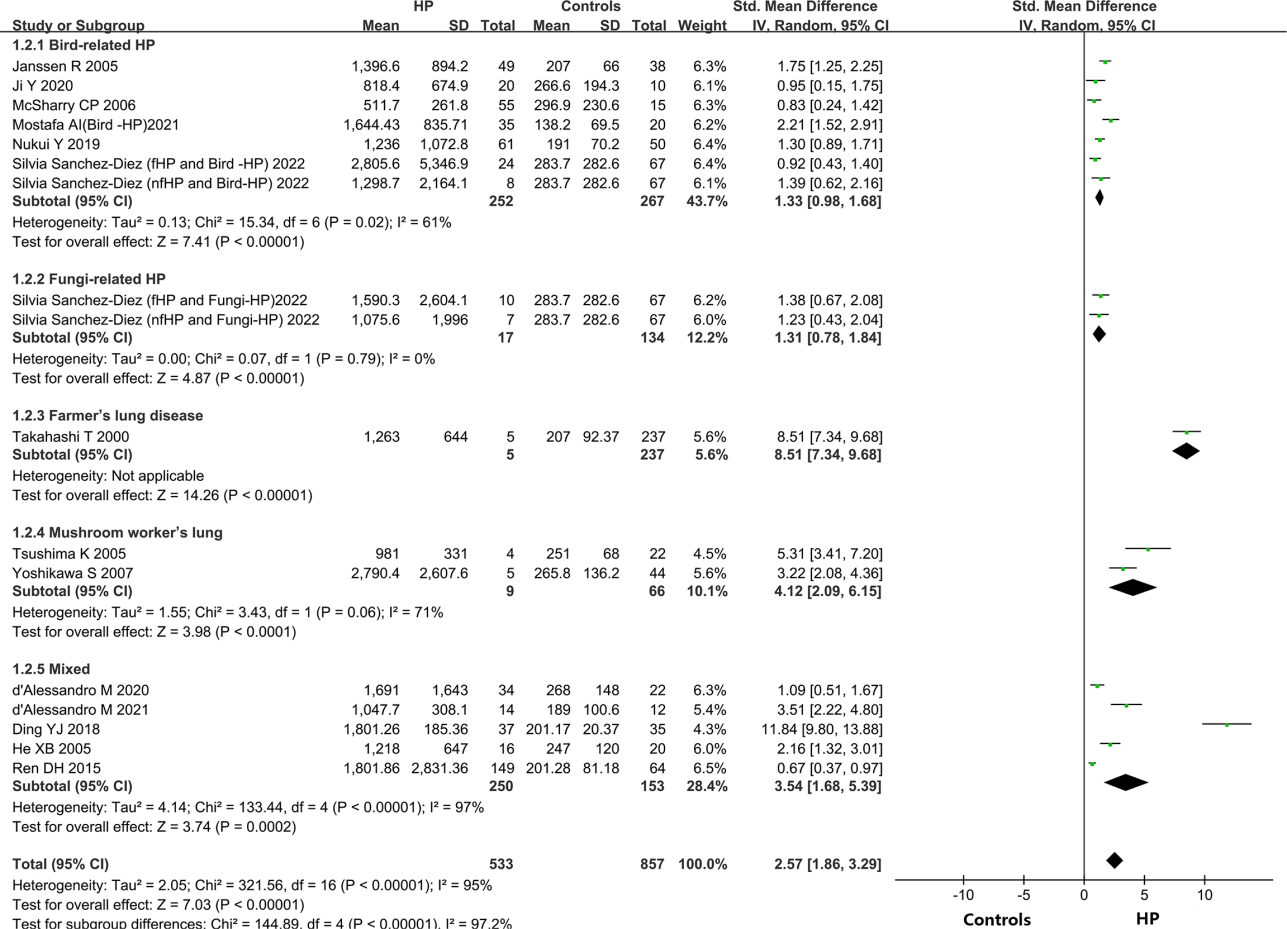
Subgroup analysis depended on different allergenic sources.

Different types of allergens to which patients are exposed can lead to different types of HP. Some of the relevant studies reported the association between HP and KL-6 concentrations due to various allergens, namely bird-related HP, fungi-related HP, HP due to Actinomyces thermophilus (also known as farmer’s lung), and mushroom-related HP (also known as mushroom lung). Subgroup analysis was performed according to HP allergens. Seven studies (9, 24–28) provided data on the concentrations of KL-6 in patients suffering from bird-associated HP versus healthy individuals, and the results revealed that the KL-6 expression was considerably higher in all patients with bird-associated HP compared to the healthy group (SMD = 1.33, 95% CI = 0.98-1.68, I^2^ = 61%, P < 0.0001); Two studies provided data on the concentrations of KL-6 in patients with fungi-related HP versus healthy individuals, and the results indicated that KL-6 concentrations were significantly higher compared to the healthy controls(SMD = 1.31, 95% CI = 0.78-1.84, I^2 =^ 0%, P < 0.0001); 2 studies (30, 31) were on mushroom lung patients versus healthy individuals, suggesting that the expression of KL-6 was substantially higher in mushroom lung patients in comparison to the healthy individuals (SMD = 4.12, 95% CI = 2.09-6.15, I^2^ = 71%, P < 0.0001). Five studies (19–23) were classified as a mixed group due to the lack of strict allergen source differentiation. The findings revealed that the concentrations of KL-6 in the mixed group of HP patients were considerably higher as compared to healthy individuals (SMD = 3.54, 95% CI = 1.68-5.39, I^2^ = 97%, P = 0.0002) (**Table 3** and **Figure 3**). Data from one study (29) revealed a higher KL-6 expression level in patients with farmer’s lung as compared to healthy individuals (1263 ± 644 vs. 207 ± 92.37 U/ml, p < 0.001). Quite notably, one article (9) reported that KL-6 concentrations were significantly higher in the serum of HP patients exposed to birds with fibrotic patterns than in healthy controls (2805.6 ± 5346.9 vs. 283.7 ± 282.6 U/ml, P<0.0001), and serum KL-6 concentrations were also significantly higher in fibrotic HP patients exposed to fungi compared with the healthy controls (1590.3 ± 2604.1 vs. 283.7 ± 282.6 U/ml, P<0.0001). Additionally, similar findings were found in the non-fibrotic HP group (nfHP and Bird-HP vs. healthy controls: 1298.7 ± 2164.1 vs. 283.7 ± 282.6 U/ml, P<0.0001; nfHP and Fungi-HP vs. healthy controls: 1075.6 ± 1996 vs. 283.7 ± 282.6 U/ml, P<0.0001).

**Table 3:** Subgroup analyses of the association between KL-6 and HP in this meta-analysis.

Subgroup	NO. of studies	Sample			Standard mean difference (95% CI)	P for Heterogeneity	I^2^	P Value between groups
		HP	Controls					
Type of Allergen								
Bird-related HP	7	252	200		1.33 [0.98,1.68]	P=0.02	61%	P<0.0001
Fungi-related HP	2	17	67		1.31 [0.78,1.84]	P=0.79	0%	P<0.0001
FLD	1	NA	NA		NA	NA	NA	NA
MWL	2	9	66		4.12 [2.09,6.15]	P=0.06	71%	P<0.0001
Mixed	5	250	153		3.54 [1.68,5.39]	P<0.0001	97%	P=0.0002
Conditions of Pulmonary fibrosis								
Fibrotic HP	7	277	218		3.28 [1.89,4.67]	P<0.0001	96%	P<0.0001
Non-fibrotic-HP	3	39	87		1.81 [0.83,2.80]	P=0.01	77%	P=0.0003
Mixed	8	233	438		2.73 [1.53,3.93]	P<0.0001	96%	P<0.0001
Ethnic								
Caucasian	11	272	184		1.75 [1.21,2.29]	P<0.0001	84%	P<0.0001
Asian	7	277	472		4.53 [2.66,6.40]	P<0.0001	98%	P<0.0001
Specimen types								
Serum	17	529	646		2.85 [2.09,3.62]	P<0.0001	95%	P<0.0001
Plasm	1	NA	NA		NA	NA	NA	NA
Assay approach								
ELISA	13	338	471		2.24 [1.47,3.01]	P<0.0001	94%	P<0.0001
CLEIA	3	191	143		5.13 [0.35,9.9]	P<0.0001	98%	P=0.04
Mixed	2	20	42		3.62 [0.54,6.69]	P=0.003	89%	P=0.02

NA, not applicable; HP, Hypersensitivity pneumonitis; FLD, Farmer’s lung disease; MWL, Mushroom worker’s lung; ELISA, Enzyme linked-immuno-sorbent assay; CLEIA, chemiluminescence immunoassay assay.

##### 3.4.2.2 Fibrosis status

The HP can be categorized into two types: fibrotic and non-fibrotic, depending upon the imaging or histopathology. Therefore, the subjects were classified into fibrotic hypersensitivity pneumonitis (fHP) and non-fibrotic hypersensitivity pneumonitis (nfHP) groups. 7 studies (9, 19–21, 23, 27) involved fHP, in which KL-6 concentrations were substantially higher as compared to healthy individuals (SMD = 3.28, 95% CI = 1.89-4.67, I^2^ = 96%, P < 0.0001). There were three studies involving nfHP (9, 27) and it was noted that the concentrations of KL-6 were elevated in these patients as compared to normal individuals (SMD = 1.81, 95% CI = 0.83-2.80, I^2^ = 77%, P = 0.0003). Eight studies (22, 24–26, 28–31) were categorized as a mixed group due to uncritical differentiation of fibrosis status. Patients with HP in the mixed group had higher KL-6 concentrations than healthy individuals (SMD = 2.73, 95% CI = 1.53-3.93, I^2^ = 96%, P < 0.0001) (**Table 3**).

##### 3.4.2.3 Ethnicity

Based on the ethnic variations, the patients were divided into two groups: Caucasian and Asian populations. Eleven studies (9, 22–27) reported the correlation between HP patients and KL-6 concentrations in a Caucasian population, suggesting that the KL-6 concentrations were higher in HP patients as compared to normal individuals (SMD = 1.75, 95% CI = 1.21-2.29, I^2^ = 84%, P < 0.0001). Seven studies reported the association between HP patients and concentrations of KL-6 in an Asian population (19–21, 28–31) The results indicated that KL-6 concentrations were considerably higher in HP patients as compared to the control individuals (SMD = 4.53, 95% CI = 2.66-6.40, I^2^ = 98%, P<0.0001) (**Table 3**).

##### 3.4.2.4 Specimen types

Seventeen studies reported sample specimens for the detection of KL-6 as serum specimens (9, 22–24, 26–31). In serum specimens, the concentrations of KL-6 were much higher in HP patients than in healthy subjects (SMD = 2.85, 95% CI = 2.09-3.62, I^2^ = 95%, P < 0.0001) (**Table 3**). Only one study (25) reported KL-6 expression in plasma specimens, indicating that concentrations of KL-6 were considerably higher in HP patients in comparison to the healthy control group (818.4 ± 674.9 vs. 266.6 ± 194.3 U/ml, p < 0.01).

##### 3.4.2.5 Detection method

Different studies used different methods to measure KL-6 concentrations. Thirteen studies used Enzyme linked-immuno-sorbent assay (ELISA) (9, 22–29), while, three studies used Chemiluminescence immunoassay assay (CLEIA) (19, 21, 31). In the ELISA group, the concentration of KL-6 was significantly elevated in HP patients as compared to normal individuals (SMD = 2.24, 95% CI = 1.47-3.01, I^2^ = 94%, P < 0.0001). In the CLEIA group, concentrations of KL-6 were also considerably higher in HP patients as compared to healthy ones (SMD = 5.13, 95% CI = 0.35-9.90, I^2^ = 98%, P = 0.04). Two studies (20, 30) were included in the mixed group because they did not mention specific measurements and the findings indicated that the concentrations of KL-6 were much more elevated in patients suffering from HP in comparison to normal individuals (SMD = 3.62, 95% CI = 0.54-6.69, I^2^ = 89%, P = 0.02) (**Table 3**).

### 3.5 Descriptive analysis

#### 3.5.1 Differences in KL-6 concentration between patients with fibrosing allergic pneumonia and non-fibrosing allergic pneumonia

Data from two publications on KL-6 concentrations in fHP versus nfHP were analyzed to further compare KL-6 concentration levels between nfHP patients and fHP patients. Mostafa AI et al. (27) observed lower concentrations of KL-6 in fHP patients (n = 27) as compared to nfHP patients (n = 24), (1,165.00 ± 283.0 vs. 2,020.83 ± 870.77 U/ml, P = 0.02), and serum KL-6 concentrations exhibited a negative correlation with hormone dose and duration of hormone treatment. However, Silvia Sánchez-Díez et al. (9) reported higher KL-6 concentrations (2448.1 ± 4700.1 vs. 2194.6 ± 2015.5 U/ml, P = 0.0289) in fHP patients (n = 34) than in nfHP patients (n = 15) (**Table 2**).

#### 3.5.2 Variations in KL-6 concentration between patients with acute allergic pneumonia and chronic allergic pneumonia

One study on the relationship between KL-6 concentration levels in acute hypersensitivity pneumonitis (AHP) and chronic hypersensitivity pneumonitis (CHP) was included. Okamoto T et al. (32) found no statistically significant variation in concentrations of KL-6 between patients suffering from AHP (n = 35) and patients suffering from CHP (n = 57) (3350 ± 3246 vs. 1639.9 ± 1250.3 U/ml, p > 0.05) (**Table 2**).

### 3.6 Sensitivity analysis

While conducting the sensitivity analysis, every one of the 20 studies was removed one after the other, and a meta-analysis of the remaining studies was carried out at each step and the results were sequentially compared with the previous elimination. If the overall impact did not change significantly, then the included studies had high stability, and the credibility of the result was verified. If the change significantly impacted the results, then the included studies had poor stability. Based on these criteria, the results suggested that the stability was good.

### 3.7 Publication bias evaluation

**Figure 4:**
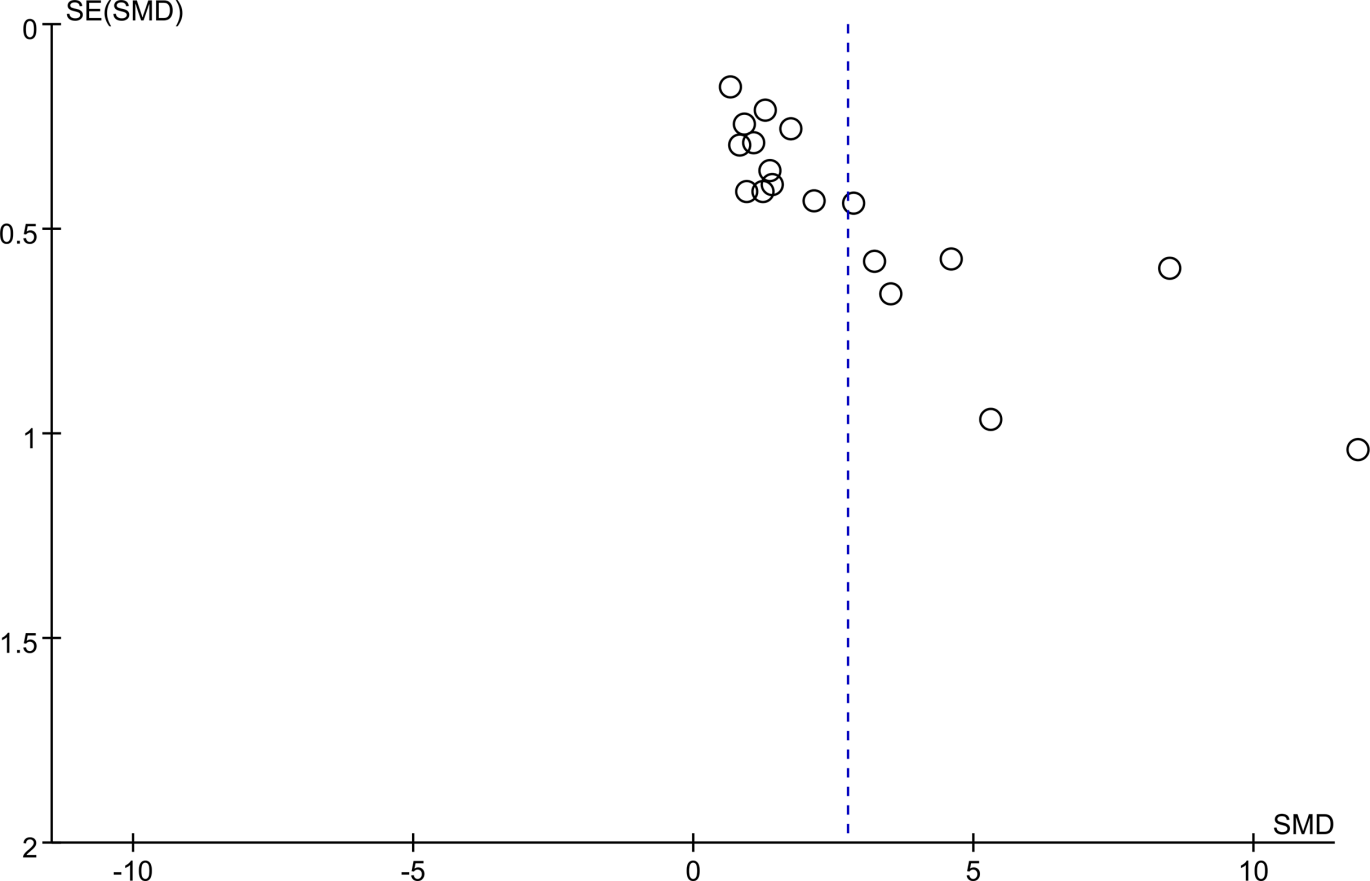
Begg’s funnel plot was to evaluate the publication bias among those included literatures about the association between KL-6 level and HP.

Egger’s regression test and Begg’s rank correlation were used to assess publication bias by constructing a funnel plot to show the relationship between the SMD and the SE of logarithmic SMD. The funnel plot for assessment of publication bias was asymmetrical, suggesting probable publication bias. Egger’s test and Begg’s test yielded similar results to the visual inspection for asymmetry of the funnel plot: Egger P <0.05; Begg P < 0.05. (**Figure 4**).

### 3.8 Bioinformatic analysis

**Table 4** Summarized information from 3 genome-wide gene expression datasets involving HP patients. In the GSE47460 dataset, the expression of KL-6 was found to be higher in lung tissues of HP patients as compared to normal lung tissues (14.21 ± 0.66 vs. 13.98 ± 0.44, t = 3.207, P = 0.002). In the dataset of GSE150910, the KL-6 expression level was higher in HP patients’ lung tissues as compared to normal lung tissues (819.39 ± 457.32 vs. 624.67 ± 296.59, t = 3.496, P = 0.001) (**Supplementary Figures 1A, B**). The KL-6 was found to be expressed mainly in type II alveolar epithelial cells, according to the single-cell sequencing dataset GSE135893, and its expression was significantly upregulated in these cells of the HP patients (P<0.001) (**Figures 5A–C**). Additionally, KL-6 mRNA expression was negatively associated with DLCO% predicted value (r = -0.452, P = 0.012) and FEV1% predicted value (r = -0.396, P = 0.032) (**Figures 6A, B**).

**Table 4:** Summary of those 3 genome-wide gene expression datasets involving HP patients.

		HP		Control				
GSE No.	Sample number (HP/Control)	Ages(mean±sd)	Sex(M/F)	Ages(mean±sd)	Sex(M/F)	Type of HP	Platform	Authors
GSE47460	30/108	57±11.09	9/21	63.62±11.35	49/59	NA	GPL6480 and GPL14550	Kim et al
GSE150910	82/103	59.4±10.6	39/43	59.9±10.2	44/56	CHP	Illumina Nova Seq 6000	Furusawa et al
GSE135893	3/10	NA	NA	NA	NA	CHP	Illumina NextSeq 500, Illumina HiSeq 4000, Illumina NovaSeq 6000	Habermann et al

CHP, Chronic Hypersensitivity Pneumonitis; NA, not applicable; M, Male; F, Female.

**Figure 5:**
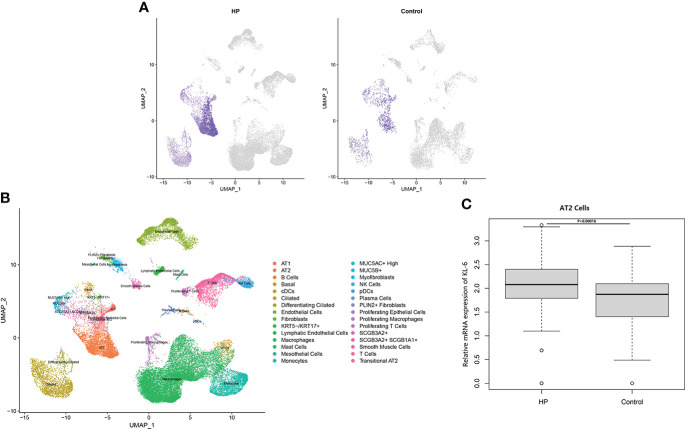
The scRNA-seq analysis in the GSE135893 (HP =3, control=8). **(A)** Feature plot of KL-6 in HP patients and controls. **(B)** Cell type annotation. **(C)** The different expressive analysis of KL-6 between HP and controls in AT2 cells.

**Figure 6:**
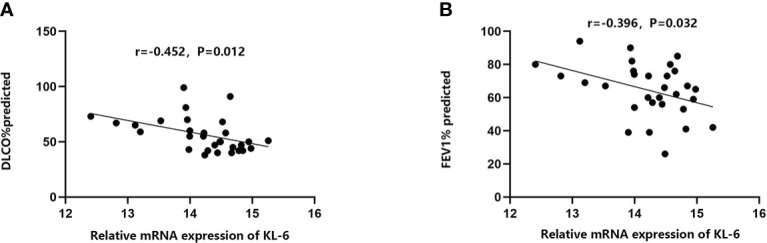
The relationships between KL-6 mRNA levels and lung function in GSE47460. **(A)** KL-6 mRNA levels were negatively correlated with DLCO% predicted (r=-0.452, P=0.012); **(B)** KL-6 mRNA levels were negatively correlated with FEV1% predicted (r=-0.396, P=0.032).

## 4 Discussion

This study revealed that the KL-6 concentrations in the blood of allergic pneumonia patients were higher in comparison to healthy individuals, which was verified in the meta-analysis of various subgroups. Elevated KL-6 concentrations may have a quantitative role in differentiating the severity and typing of HP. In both Caucasian and Asian populations, concentrations of KL-6 were much higher in HP-suffering patients in comparison to healthy individuals, and the increase was even more pronounced in the Asian population with HP, which may be associated with different living environments and the differences in immune responses to allergens in different ethnic groups. The results of bioinformatics analysis suggested that KL-6 concentrations in HP lung tissue and type II alveoli were higher in comparison to healthy control groups, and higher KL-6 concentrations displayed a negative correlation with DLCO% predicted and FEV1% predicted indexes in HP patients. Okamoto T’s (32) study revealed that serum KL-6 concentrations in chronic HP were significantly higher during episodes of acute exacerbation than 1 month before acute exacerbation. Therefore, Serum KL-6 concentrations might be useful for the diagnosis and management of chronic HP.

Sustained high concentrations of KL-6 were closely linked to the progression of interstitial lung disease (33, 34). Additionally, concentrations of KL-6 are closely related to a substantial decrease in FVC% and DLCO% (35, 36). Progressive allergic pneumonia is often accompanied by extensive ground glass shadows and elevated serum KL-6 concentrations on HRCT, and pathologic biopsies demonstrate that the diagnosis of progressive allergic pneumonia is consistent with diffuse alveolar injury. Therefore, elevated KL-6 concentrations are consistent with the degree of extensive alveolar injury, which in turn may improve the diagnosis of HP. Rapidly elevated KL-6 concentrations suggest the need for early differentiation between fibrotic HP and non-fibrotic HP.

**Figure 7:**
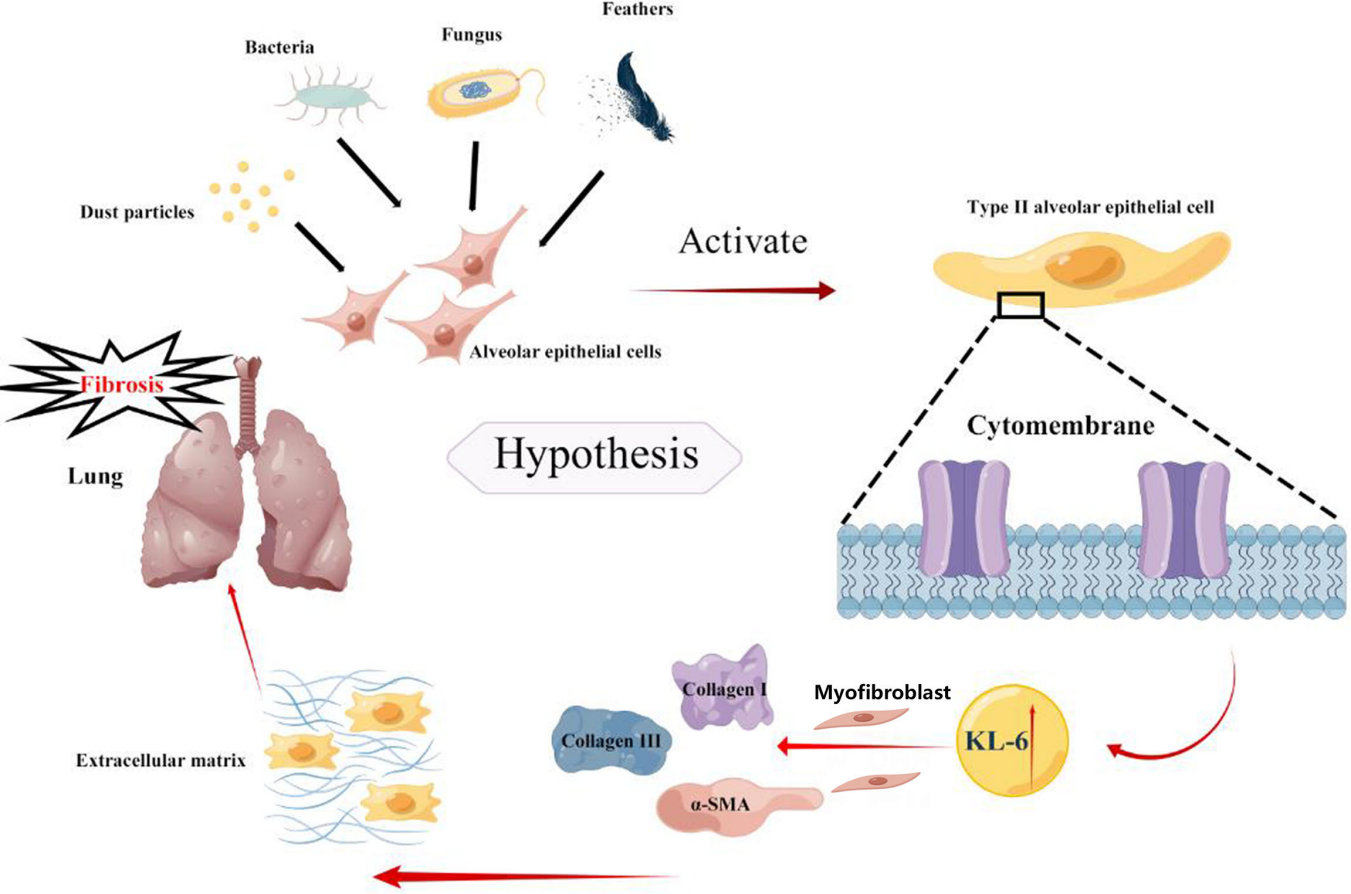
The possible potential mechanism of KL-6 in HP.

Based on previous studies, the present meta-analysis concludes that higher KL-6 concentrations were detected in patients with HP as compared to normal individuals and the increase was even more pronounced in patients with fibrotic HP. There are two primary reasons which explain the elevation in the KL-6 concentrations that can efficiently differentiate the severity and various states of HP. First, the types of pathological injury in HP are diverse, with the most common types being pulmonary vascular epithelial injury and diffuse alveolar epithelial cell injury. Chronic fibrosis formation is the primary pathological mechanism of alveolar epithelial cell injury (37).KL-6 is a glycoprotein, with the MUC1 gene as its main regulator, and it is highly expressed on the surface of regenerating type II alveolar epithelial cells (38, 39), a view validated by the single-cell sequencing dataset in the present study. When lung mesenchymal or parenchymal epithelial cells are damaged, the basement membrane integrity of lung capillaries is disrupted, leading to increased capillary permeability in the lung. In order to repair the damaged type I alveolar epithelial cells, type II alveolar epithelial cells proliferate significantly, leading to an increase in secreted KL-6 entering the blood *via* the lungs (8). Additionally, HP, an allergic disease, is characterized by a systemic inflammatory response syndrome. Activation of inflammatory factors and KL-6 expression in type II alveolar epithelial cells may lead to progressive changes in lung imaging and fibrotic changes in the lung suggesting more severe lung injury. Xu L (40) observed that damaged alveolar epithelial cells release KL-6 to initiate fibroblast to myofibroblast differentiation, increase type I and type III collagen expression, and inhibit HGF production. Therefore, KL-6 is an important molecule involved in epithelial-mesenchymal transition and fibrogenesis in interstitial lung disease, Patients with fibrotic HP have higher KL-6 concentrations. We speculated that KL-6 could promote the accumulation of extracellular matrix leading to fibrosis in the lung of HP patients (**Figure 7**). Elhai M (41) found elevated serum KL-6 concentrations while assessing the severity of IPF. A cross-sectional study by Sánchez-Díez S (9) also suggested higher concentrations of KL-6 were observed in serum and sputum of fibrotic HP as compared to patients with non-fibrotic HP. However, the study conducted by Mostafa AI (27) revealed higher serum KL-6 concentrations in non-fibrotic HP compared to fibrotic HP. The reason for the inconsistency between the results of Mostafa AI’s and our meta-analysis could be due to the small sample size of the Mostafa AI study (fibrotic HP: 27 cases, non-fibrotic HP: 24 cases). Furthermore, the population included in their study was mainly female patients, and the gender factor may have caused bias in the results. Previous studies did not have a uniform definition for the division of acute and chronic HP (42), therefore, some patients may also present with pulmonary fibrosis manifestations in a short period. Defining acute and chronic HP by the time of onset is incomplete for the diagnosis of the disease, therefore, expanded samples are needed to verify the possible effects of KL-6 concentrations in the classification of fibrotic and non-fibrotic HP.

The bioinformatics findings of this research imply that elevated KL-6 concentrations are also of predictive value for the assessment of pulmonary function in HP patients, especially for DLCO% and FEV1% predicted values. In the current study, the correlation coefficients of KL-6 with DLCO% and FEV1% predicted values were -0.452 and -0.396, respectively, in agreement with the findings of Sánchez-Díez S (9). Sánchez-Díez S also concluded that serum concentrations of KL-6 displayed a negative correlation with total lung capacity (r = -0.485; P = 0.0103) and diffusing capacity of the lungs for carbon monoxide (r = -0.534; P = 0.0002) at 12 months. Another prospective cohort study (43) also found that DLCO would decrease by 15% when the cut-off value of KL-6 was 1472 U/ml. Another case-control study reported that increased serum concentrations of KL-6 were also negatively associated with FVC% and FEV1% (44). In interstitial lung disease, high-resolution CT ground glass shadow score and serum concentrations of KL-6 are independent risk factors for progressive interstitial lung disease. Increased concentrations of KL-6, to some extent, might correlate with progressive lung damage monitored by HRCT under inflammatory storm (45). Inflammatory factors can promote the development of pulmonary fibrosis in patients with HP, as subacute and chronic HP are primarily T cell-mediated immune responses, with boosted T cell migration in the lungs and the development of characteristic T lymphocytic alveolitis (46, 47). Furthermore, type IV metaplasia in chronic HP also forms granulomas and interstitial fibrosis through an immune response involving T cells and macrophages (5). From a total of 15 literatures, eight literatures (9, 22–24, 26, 30–32) provided information on the smoking history and current smoking behavior of participants. Unfortunately, due to missing data regarding KL-6 concentration in different smoking status groups, we were unable to carry out this subgroup analysis. Arima K et al. (48) indicated that no difference was found in serum anti-T. cutaneum antibody activities or the bronchoalveolar lavage lymphocyte phenotypes for smoking and nonsmoking summer-type hypersensitivity pneumonitis (SHP) patients. They concluded that cigarette smoking had a suppressive effect on the outbreak of SHP, but smoking caused no further suppression after the disease was established. Dangman KH et al. (49) implemented a retrospective study targeting metalworkers with HP, and researchers found that the development of hypersensitivity pneumonitis is forestalled in cigarette smokers. Kobayashi H et al. (50) reported that serum KL-6 was unaffected by smoking in COPD patients and could not predict preclinical lung damage induced by smoking. However, the above studies had the limitations of a short follow-up time, and a relatively small sample size. Studies with larger sample sizes are needed to confirm whether smoking status could correlate with the type of HP or KL-6 levels.

Elevated KL-6 concentrations may increase the morbidity and mortality of interstitial lung disease (45). A Chinese cohort study reported that KL-6 concentrations were substantially higher in patients with progressive ILD as compared to non-progressive ILD. When corrected for other covariates, serum KL-6 concentration was an independent risk factor for predicting patient death (51). A prospective multifactorial analysis of systemic sclerosing interstitial lung disease (52) revealed that serum KL-6 was a reliable predictor of the progression of end-stage lung disease and these patients have extremely poor lung function, often require continuous oxygenation, and may die within a short period. The decreased survival of patients may be due to progressive interstitial lung changes following damage to lung tissue and the release of large amounts of KL-6 from alveolar epithelial cells. Hanzawa S (53) in his study noted that relative variations in the concentration of serum KL-6 were associated with fibrotic Bird-related Hypersensitivity Pneumonitis (BHP) in hypersensitivity pneumonitis patients. The relative variation in the concentrations of KL-6 (greater than vs. less than a 10% decrease) was notably correlated with survival in a stratified analysis (73.9 vs. 34.9 months; P = 0.04). This study did not do a combined analysis of the prognostic value of KL-6 for HP because of the small body of literature available on this topic. However, this study evaluated the association between KL-6 and pulmonary function, which displayed a negative correlation with DLCO% predicted and FEV1% predicted in patients with HP. Several prior studies identified prognostic factors in chronic HP, with lower FVC%, FEV1, and foveal changes on HRCT associated with shorter survival time (54, 55). Indeed, some severe diseases such as ARDS and septic shock usually also present a sharp spike in serum concentrations of KL-6 (56). Thus, KL-6 concentrations may also be closely related to the prognosis of HP, with an intrinsic link between impaired lung function and high concentrations of KL-6.

Pre-existing HP is categorized into acute, subacute, and chronic HP according to the pace of the onset (57). However, there are no uniform criteria for the temporal identification of acute, subacute, and chronic HP, therefore, subgroup analysis according to the pace of onset of HP was not performed in this study. American Thoracic Society (2020) classified HP into non-fibrotic and fibrotic HP according to the availability of fibrotic features on imaging or histopathology (10). Therefore, a subgroup analysis of HP was carried out in accordance with these subtypes. The results still suggested that concentrations of KL-6 were higher in HP patients in comparison to normal individuals, whereas, the elevated KL-6 concentrations were even more pronounced in patients suffering from fibrotic HP, suggesting that KL-6 might predict the pulmonary fibrosis status in HP patients. Additionally, increased concentrations of KL-6 might act as an immunological biomarker to differentiate the source of allergens in HP patients, as KL-6 differs in patients with bird-associated allergic pneumonia, fungal-associated allergic pneumonia, and actinomycete-associated allergic pneumonia (32). Therefore, this meta-analysis will provide a reference for future calculation of the diagnostic value of KL-6 in specific HP classifications.

Testing for KL-6 concentrations in serum or plasma has several advantages. Firstly, KL-6 concentrations can be used as an initial assessment of a patient’s condition if the patient refuses to undergo invasive testing or surgery. Secondly, imaging may lag behind current changes in clinical practice, resulting in delays in diagnosis, treatment, or assessment of the condition. To overcome these deficiencies, physiological indicators have been proposed clinically to help determine prognosis, including the GAP and CPI indexes. Currently, clinical serum markers, such as KL-6, have not been able to replace existing physiologic measurements to predict patient prognosis, however, each of these assays and methods has its limitations. Combined tests based on physiological indices, lung imaging, and serum markers are more accurate in predicting the prognosis of patients suffering from HP.

In the research carried out by Mostafa AI. et al. (27, 32, 58), serum concentrations of KL-6 decreased significantly in patients after aggressive steroid and antifibrotic therapy. Here are the two main reasons for this shift: first, both antifibrotic and immunosuppressive drugs attenuate the inflammatory response of type II alveolar epithelial cells (46), thus, the reduction in inflammatory exudation eventually leads to a decrease in the release of KL-6 (59–62). Secondly, HP treatment can stop the progression of chronic fibrosis, inhibit the over proliferation of pulmonary vascular epithelial cells, and to some extent modulate the hyperfunction of the human immune system (63). This therapeutic effect clearly attenuates other impairments of HP and the concentration of KL-6 will progressively decrease with appropriate treatment. Therefore, monitoring of serum concentration of KL-6 in patients with HP can help in the assessment of patient outcomes.

Although stratified analyses have been carried out to every extent possible, the present meta-analysis still has certain shortcomings. First, the lack of randomized controlled trials or prospective cohort studies with large samples may reduce the credibility of the results of this study’s analysis. Second, the classification criteria for HP varied among studies, leading to great heterogeneity and publication bias. Third, the included studies were from different countries and regions, and different geographical locations, environmental factors, and lifestyles are the sources of heterogeneity. Fourth, HP infection rates are higher in women than in men, which may be related to the etiology or pathogenesis of the disease itself (64), therefore, gender-based subgroup analysis is not feasible due to the lack of gender-specific studies of HP patients. Therefore, there is an urgent need for more detailed, quality research and long-term follow-up studies.

## 5 Conclusion

In this study, we quantitatively compared KL-6 concentrations in allergic pneumonia and derived the SMD of KL-6 between various groups. We conclude that concentrations of KL-6 are higher in patients with HP as compared to healthy individuals, and the detection of KL-6 concentrations can assist in evaluating the severity of HP and the effectiveness of its treatment. However, most of the original studies were conducted in Asia or Europe. Therefore, we hope that future studies might include African and Latin American populations. The present publication can guide studies in the future, for instance, the dose-response key between increased concentrations of KL-6 and HP progression. The research conducted in the future might be directed towards concentration cut-off values of KL-6 for the diagnosis of HP to prompt physicians to intervene earlier in progressive HP.

## Data availability statement

The original contributions presented in the study are included in the article/**Supplementary Material**. Further inquiries can be directed to the corresponding author.

## Author contributions

JH developed the research idea, performed data collection, performed data analysis, performed the scRNA-seq analysis, prepared the first manuscript draft, refined the research idea, and edited the manuscript. JZ and XR validated data collection, developed the research idea, and proofread the article. All authors listed have made a substantial, direct, and intellectual contribution to the work and approved it for publication. All authors contributed to the article and approved the submitted version.

## Funding

This work was supported by Sichuan Medical Research Project Foundation (S21054).

## Conflict of interest

The authors declare that the research was conducted in the absence of any commercial or financial relationships that could be construed as a potential conflict of interest.

## Publisher’s note

All claims expressed in this article are solely those of the authors and do not necessarily represent those of their affiliated organizations, or those of the publisher, the editors and the reviewers. Any product that may be evaluated in this article, or claim that may be made by its manufacturer, is not guaranteed or endorsed by the publisher.

## Supplementary material

Supplementary Figure 1 Expression of KL-6 lung tissue in patients with HP in GEO datasets. **(A)** GSE47460;**(B)** GSE150910.
