# Supplementary figures and images for: Krebs von den lungen-6 as a clinical marker for hypersensitivity pneumonitis: A meta-analysis and bioinformatics analysis
Source: Front Immunol. 2022 Nov 30;13:1041098. doi: 10.3389/fimmu.2022.1041098 (PMC9748086; doi:10.3389/fimmu.2022.1041098)

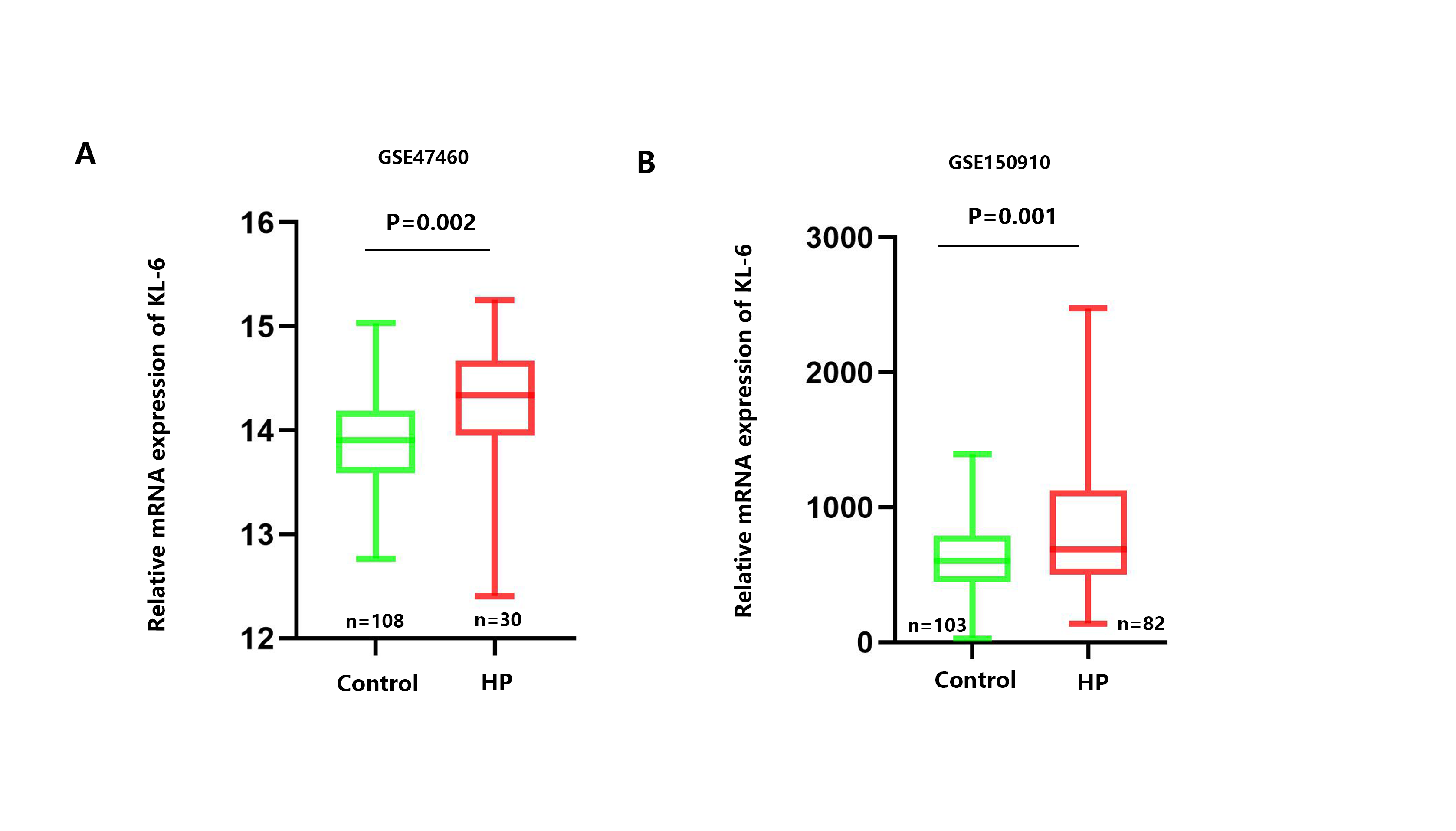

Supplement: Supplementary Figure 1 — Expression of KL-6 lung tissue in patients with HP in GEO datasets. (A) GSE47460;(B) GSE150910. [file Image_1.tif]
